# Chemistry in Transformation: A European Moment

**DOI:** 10.1002/anie.202526116

**Published:** 2025-12-19

**Authors:** Javier García‐Martínez

**Affiliations:** ^1^ Departamento de Química Inorgánica, Laboratorio de Nanotecnología Molecular Universidad de Alicante Carretera San Vicente s/n Alicante 03690 Spain

**Keywords:** Chemical industry, Decarbonization, Digitalization, Industrial competitiveness, Responsible innovation

## Abstract

Chemistry stands at a pivotal juncture, facing unprecedented expectations to decarbonize, enable circularity, and sustain essential sectors, while remaining globally competitive. Europe embodies these pressures acutely. Although the region excels in frontier research, structural disadvantages such as high energy and feedstock costs, fragmented regulation, slow permitting, and constrained capital limit its capacity to translate scientific leadership into industrial success. The energy crisis of 2022 exposed long‐standing vulnerabilities, triggering capacity closures in foundational value chains such as ammonia and olefins and accelerating the erosion of Europe`s market share. Yet chemistry`s transformation is driven not only by economic asymmetry, but by deeper shifts: the urgency of decarbonization, the need for molecular circularity, and the integration of digital technologies into discovery and manufacturing. Breakthroughs in electrification, advanced catalysts, CO_2_ conversion, self‐driving laboratories, and AI‐enabled synthesis offer pathways to both competitiveness and sustainability, but their impact depends on coordinated infrastructure, stable policy, and risk‐tolerant finance. Crucially, this moment also demands responsibility and resilience: chemistry must be circular by design, transparent in its data, and robust enough to withstand shocks, diversify feedstocks, and scale technologies reliably. Europe`s leadership will hinge on embedding these principles into how chemistry is imagined, taught, and deployed.

## Graphical abstract

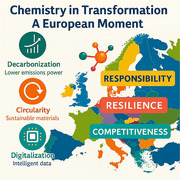

Europe’s chemical industry faces a defining moment. Decarbonization, circularity, and digitalization are reshaping competitiveness amid high energy costs and investment challenges. Integrating responsible and resilient chemistry with innovation, infrastructure, and policy is essential to translate scientific leadership into sustainable industrial renewal and long‐term industrial leadership.

Never before has chemistry been asked to shoulder so many expectations at once. It is called on to simultaneously decarbonize energy and materials, make resource use more circular, and underpin safe and reliable food and healthcare, all while continuing to deliver returns and defend its position in an increasingly competitive global marketplace. Europe illustrates this tension with exceptional clarity: despite its strength in frontier research, it faces rising constraints on energy costs, industrial competitiveness, technology scaling, and the capacity to translate discovery into innovation.^[^ ^1^, ^2^ ^]^ At the same time, many of the dynamics visible in Europe mirror wider global forces. Chemistry is being reshaped everywhere, not only by scientific discoveries but by geopolitical tension, new tariff regimes, fragmented regulatory environments, shifting supply chains, and an increasingly unstable global trading system. In such a world, chemistry's transformation depends on coupling scientific excellence with the ability to scale new technologies and build the infrastructure they require. Incremental improvements will not suffice: chemistry must be reimagined to meet global demand while sharply reducing its environmental footprint.^[^ ^3^, ^4^ ^]^

## Europe's Structural Competitiveness Stress Test

The energy crisis of 2022 acted as a trigger, exposing and amplifying structural vulnerabilities that had been accumulating for more than a decade. Europe's chemical industry had long faced higher energy and feedstock costs relative to global competitors, slower and more uncertain permitting, and increasing pressure from new capacity concentrated in regions with lower production costs.^[^ ^1^, ^2^, ^5^ ^]^ The crisis transformed these long‐standing disadvantages into acute competitive stress. In the period that followed, European chemical production entered a period of depressed demand and reduced utilization, with operating rates around 75%. Since 2023, announcements have been made to close more than 11 million tonnes of chemical production capacity, largely in upstream chains such as ammonia, olefins, and aromatics.^[^ ^1^, ^5^, ^6^ ^]^ These closures far exceed normal cyclical variation, and once large capital‐intensive units are shuttered, they are rarely restarted, given the reinvestment required, the cost of capital, and regulatory complexity.^[^ ^5^, ^6^ ^]^

**Figure 1:**
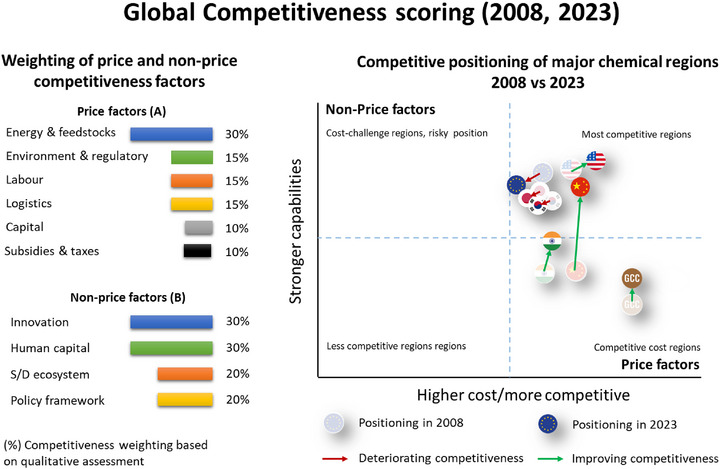
Comparative competitiveness scoring of major chemical regions in 2008 and 2023, integrating price factors (energy, labor, logistics, capital) and non‐price factors (innovation, human capital, ecosystem strength, policy framework). The analysis shows significant regional repositioning, with Europe's relative decline driven primarily by worsening cost factors. Adapted from Competitiveness of the European Chemical Industry, Ref. 5.

The demand slowdown has been compounded by a persistent structural cost gap (see Figure 1). Even after the peak of the crisis, European gas and electricity prices remained roughly two to three times higher than those in the United States, while China and India benefited from discounted crude and the United States from abundant low‐cost ethane.^[^ ^1^, ^6^, ^7^ ^]^ These asymmetries disproportionately affect energy‐intensive value chains such as ammonia, olefins, methanol, and chlor‐alkali, the foundational building blocks that underpin many of Europe's downstream specialties, polymers, and advanced materials. Over the longer term, Europe's global market share in chemicals has declined by roughly eleven percentage points since 2008, and both output and capacity utilization remain below long‐run averages.^[^ ^1^, ^5^ ^]^

**Figure 2:**
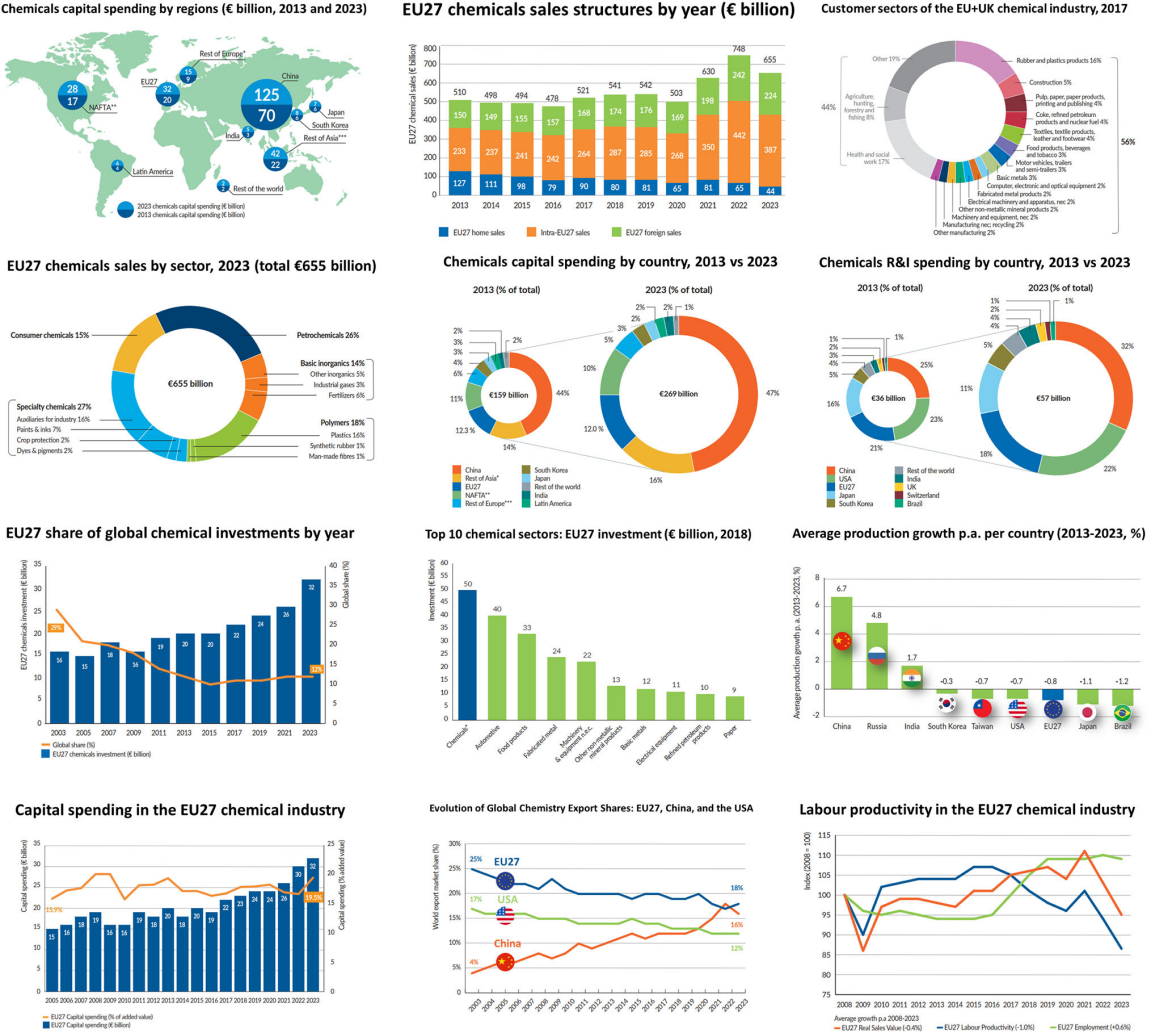
Key indicators illustrating Europe's declining competitiveness in the global chemical industry. The data highlight sustained cost disadvantages, falling investment, and shrinking export shares, contrasted with strong growth in China and other regions. Adapted from *Cefic, Facts & Figures 2024: The European Chemical Industry in the Global Competitive Landscape*, Ref. 6.

Regulation, although fundamental to Europe's social and environmental model, also contributes to competitive pressure. The policy landscape has become more complex and fast‐moving, combining ambitious climate and environmental objectives with evolving requirements for product safety, circularity, and reporting.^[^ ^2^, ^8^ ^]^ While these regulations reflect legitimate societal priorities, the proliferation of regulatory initiatives and their uneven implementation across Member States, combined with comparatively high compliance costs, creates uncertainty for long‐term investment and increases the risk of production shifting offshore.^[^ ^2^, ^5^ ^]^

These trends intersect with deeper investment and innovation bottlenecks. Europe's share of global chemical capital expenditure has fallen over the past decade, while major expansions in Asia and the Middle East have delivered new world‐scale assets with high integration and efficiency.^[^ ^5^, ^6^ ^]^ Many European plants are older and smaller than their global counterparts. Despite its exceptional scientific strength, Europe continues to struggle in converting research leadership into industrial competitiveness.^[^ ^4^, ^8^ ^]^ The deficit lies not in ideas but in the conditions required to deploy them at scale: predictable permitting, risk‐tolerant finance, FOAK/NOAK infrastructure, and coordinated cross‐border projects (Figure 2).

## Strategic Drivers Reshaping the Chemical Sector: Decarbonization, Circularity, and Digitalization

Decarbonization is one of the most significant forces reshaping the chemical enterprise. The primary chemical sector, including ammonia, methanol, ethylene, propylene, benzene, and toluene, anchors global value chains and accounts for a substantial share of global industrial CO_2_ emissions.^[^ ^7^, ^9^ ^]^ Steam cracking, ammonia synthesis, and methanol reforming together contribute a major portion of this footprint. Today, only a small fraction of global capacity operates via low‐emission routes, and meeting net‐zero trajectories by mid‐century would require trillions of dollars in additional investment encompassing renewable power, low‐carbon hydrogen, and CO_2_ capture, transport, and storage infrastructure.^[^ ^7^, ^9^ ^]^

Yet technologies are advancing rapidly. Electrified steam crackers, high‐efficiency catalysts, CO_2_‐to‐chemicals pathways, and integration of biomass and waste‐derived feeds demonstrate that deep emissions reductions are technically feasible.^[^ ^4^, ^7^, ^9^ ^]^ Yet without coordinated infrastructure planning, long‐term power agreements, and stable carbon policies, these solutions will remain confined to isolated demonstrations.

The circular transition illustrates both the ambition and the limitations of Europe's current model. Circularity is not merely an end‐of‐life correction but a design principle that influences how molecules, materials, and products are conceived, synthesized, used, and reintegrated.^[^ ^8^ ^]^ Crucially, there can be no genuine circular economy without circular chemistry: every loop of recovery and reuse ultimately depends on chemical transformations.^[^ ^10^ ^]^ Companies across Europe have initiated pilots in recycling, bio‐based feedstocks, and CO_2_‐derived intermediates, but economic incentives, infrastructure, and regulatory clarity lag behind scientific capability.^[^ ^5^, ^8^, ^9^, ^10^ ^]^

The third major driver of transformation is digitalization, particularly the intelligent use of data in discovery, manufacturing, and lifecycle management. Autonomous laboratories, self‐driving experimentation, and AI‐enabled synthesis and design now allow iterative exploration of chemical space at unprecedented speed and scale.^[^ ^11^, ^12^, ^13^ ^]^ Machine‐learning models trained on multimodal experimental and computational data, coupled with high‐throughput experimentation and advanced spectroscopic characterization, enable more efficient, reproducible, and targeted discovery. In production, digital twins, predictive control systems, and advanced analytics now allow processes to be optimized in real time, improving energy and resource efficiency and strengthening environmental performance.^[^ ^12^, ^14^, ^15^ ^]^ These capabilities align with European efforts to treat advanced materials and digitalization as strategic assets for industrial leadership.^[^ ^16^ ^]^ Yet the value of this transformation is still only partially captured. Fragmented data architectures, limited interoperability, cybersecurity concerns, and shortages of digitally skilled personnel prevent these tools from scaling across sites and value chains.^[^ ^15^ ^]^ Unlocking their full potential will require shared data standards, interoperable digital infrastructure, and coordinated upskilling across Europe's industrial clusters.^[^ ^15^, ^16^ ^]^

Despite these headwinds, Europe retains formidable strengths. It hosts world‐class universities, research centers, and infrastructures. Yet Europe still struggles to convert its scientific excellence into large‐scale industrial projects. This gap concerns technology transfer, entrepreneurship, risk finance, regulatory agility, and industrial partners capable of scaling novel technologies. The IUPAC Chemistry Entrepreneurship initiative responds directly to this need, equipping chemists with skills for commercialization, intellectual property, investment readiness, and scale‐up planning.^[^ ^17^ ^]^ In parallel, the IUPAC Top Ten Emerging Technologies in Chemistry provides an annual horizon scan of innovations with the strongest potential to transition from laboratory research to market deployment.^[^ ^18^ ^]^ By identifying and communicating these emerging opportunities early, the initiative helps direct attention, investment, and strategic support toward the areas where chemistry can deliver the greatest societal and industrial benefit.

## Responsibility, Resilience, and the Evolving Purpose of Chemistry

A growing set of international initiatives launched this past year underscores a shared conviction: chemistry's future depends on embedding responsibility and resilience at the core of its transformation. Among them, the Stockholm Declaration on Chemistry for the Future sets out a vision of a discipline aligned with sustainability, equity, and global collaboration and, above all, issues a call to action. It urges scientists, industry, educators, students, and policymakers to move beyond incremental change and work together to redesign chemistry so that it minimizes harm, is circular by design, transparent in its data, and explicitly oriented toward long‐term societal value.^[^ ^19^, ^20^ ^]^

The IUPAC Guiding Principles turn this vision into a clear professional standard, highlighting integrity, transparency, inclusivity, safety, environmental responsibility, and the commitment to collaborate and innovate responsibly.^[^ ^21^ ^]^ These Guiding Principles invite a shift in perspective from what chemistry can do to what chemists should do.^[^ ^22^ ^]^ They call for responsibility to be taught, practiced, and institutionalized, linking education, research, and innovation within a systemic vision. Far from limiting competitiveness, such reflection strengthens it. Industries that demonstrate credible responsibility in safety, environmental performance, and transparency consistently secure faster permitting, stronger investor confidence, and more stable market access. When responsibility is embedded in how chemistry is done, it becomes a source of competitive advantage, not a constraint. ^[^ ^21^, ^22^, ^23^, ^24^ ^]^ Building on this foundation of responsibility, the RESILIENCE Principles argue that sustainable chemistry must also be robust, able to withstand shocks, adapt to disruption, and continue supplying essential products under volatile conditions. Resilience is not an abstraction but a set of deliberate design choices. In practice, this means diversifying feedstocks, designing flexible and fail‐safe processes, building infrastructures that avoid single‐point vulnerabilities, and preparing a workforce able to anticipate and manage disruption. Resilience thus complements sustainability by ensuring that new technologies can scale reliably and continue to deliver value under volatile conditions.^[^ ^23^ ^]^

## A European Playbook for Action

Europe already benefits from assets that few regions can match. A unified regulatory framework and the free movement of people, knowledge, and technology have fostered a uniquely collaborative scientific ecosystem. Institutions such as the European Chemical Industry Council (Cefic), the European Chemical Society (EuChemS), and the European Chemicals Agency (ECHA) demonstrate how research, policy, and industry can work together within a shared space to advance common goals. Yet these strengths are offset by persistent structural weaknesses that slow deployment and scale‐up. Fragmented innovation policies, uneven energy costs, and limited access to risk‐tolerant capital continue to constrain Europe's ability to translate frontier research into globally competitive industrial capacity.^[^ ^1^, ^2^, ^5^, ^6^ ^]^

**Figure 3:**
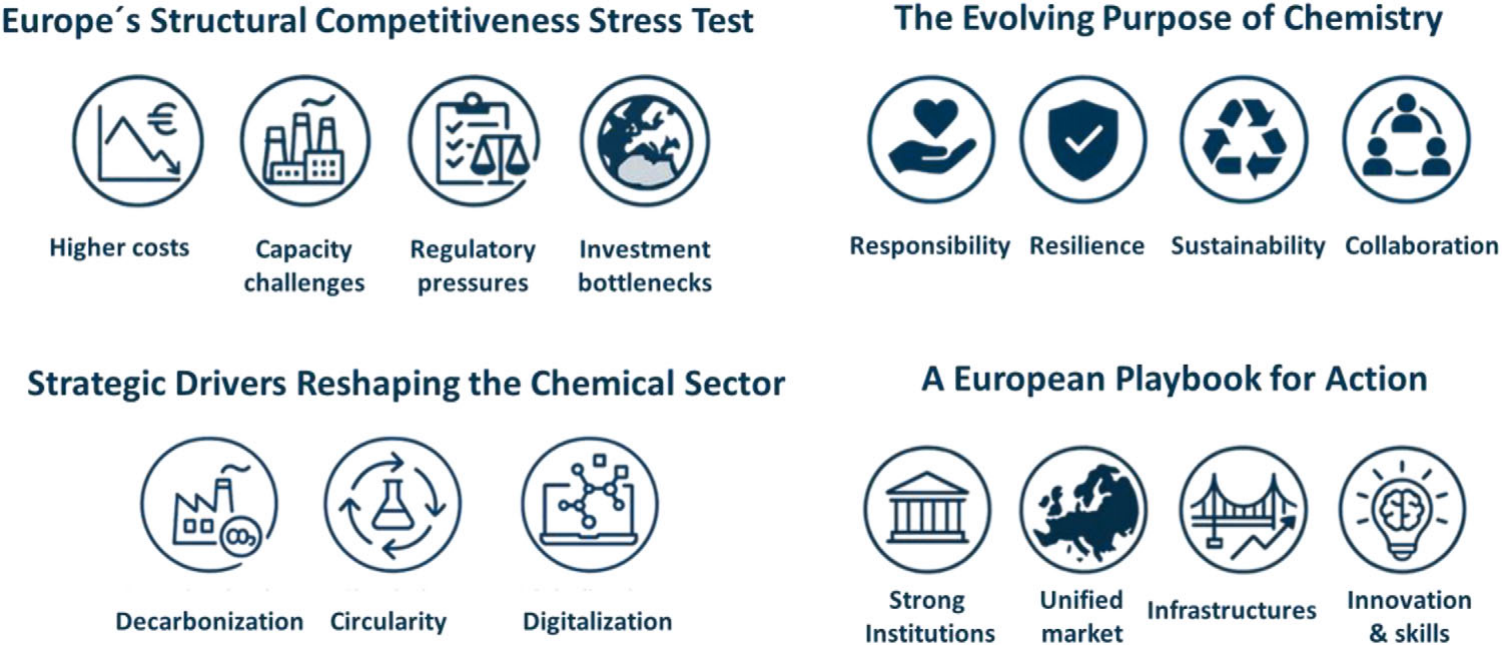
Visual summary of Europe's main structural competitiveness challenges, the key elements shaping the evolving purpose of chemistry, the three major drivers reshaping the chemical sector, and the priority areas for advancing the sector's transformation in Europe.

Europe already holds many of the elements needed to lead: world‐class science, strong institutions, and a large unified market. But these strengths only translate into real competitiveness when they are matched with predictable energy costs, faster permitting, and infrastructure that works across borders. Financing must also help promising technologies move from the lab to commercial scale. Equally important are supportive policy frameworks, clear and coherent regulation, and markets where low‐carbon and circular products can grow. Education systems should pair scientific excellence with the entrepreneurial skills needed to turn ideas into solutions. By strengthening these enabling conditions, Europe can turn its research power into the transformative capacity required for a resilient and competitive chemical industry, as summarized in Figure 3.^[^ ^2^, ^8^, ^16^, ^18^, ^24^ ^]^

The forthcoming 10^th^ EuChemS Chemistry Congress (ECC10) represents a decisive opportunity to confront these challenges and to articulate a renewed vision for the role of chemistry in Europe's competitiveness. Arriving at a complex moment, ECC10 will bring together leading researchers, emerging scientists, industry innovators, and policy stakeholders. A dedicated Industry Day will offer a platform for researchers, companies, clusters, and financial institutions to identify technological opportunities, examine deployment barriers, and develop partnerships required to scale emerging technologies across Europe. Such forums will be increasingly important for aligning scientific capability with industrial deployment and strengthening Europe's innovation ecosystem. I hope you can join us in Antwerp and contribute to shaping the next chapter of European chemistry.

## Conclusion

This is a complex moment, defined by structural transformations, geopolitical fragmentation, and the challenge of aligning economic prosperity with environmental responsibility.^[^ ^7^, ^9^, ^24^, ^25^ ^]^ Yet Europe's chemistry community has the capacity to invent rather than inherit the future. The transformation of chemistry is not a zero‐sum game: by integrating decarbonization, circularity, digitalization, responsibility, and resilience into a coherent strategy, Europe can build a chemical industry that is both sustainable and competitive. Chemistry's renewal will be measured not only by the technologies we create but by the wisdom with which we apply them, to deepen understanding, foster collaboration, and sustain life on a changing planet. Europe's scientific depth and industrial base position it uniquely to shape a future in which chemistry becomes a driver of both economic renewal and geopolitical relevance.

J. G.‐M. is Director of the Molecular Nanotechnology Lab at the University of Alicante (Spain) and served as President of IUPAC (2022–2023). Founder of Rive Technology, a company that commercialized advanced catalytic materials. He contributes to global initiatives shaping chemistry, including the Stockholm Declaration on Chemistry for the Future, IUPAC Guiding Principles of Responsible Chemistry, and RESILIENCE Principles for Chemistry.
